# Intraretinal Fluid Pattern Characterization in Optical Coherence Tomography Images

**DOI:** 10.3390/s20072004

**Published:** 2020-04-03

**Authors:** Joaquim de Moura, Plácido L. Vidal, Jorge Novo, José Rouco, Manuel G. Penedo, Marcos Ortega

**Affiliations:** 1 Centro de investigación CITIC, Universidade da Coruña, 15071 A Coruña, Spain; joaquim.demoura@udc.es (J.d.M.); jnovo@udc.es (J.N.); jrouco@udc.es (J.R.); mgpenedo@udc.es (M.G.P.); mortega@udc.es (M.O.); 2 Grupo VARPA, Instituto de Investigación Biomédica de A Coruña (INIBIC), Universidade da Coruña, 15006 A Coruña, Spain

**Keywords:** Optical Coherence Tomography, texture analysis, feature selection, computer-aided diagnosis, classification, feature analysis

## Abstract

Optical Coherence Tomography (OCT) has become a relevant image modality in the ophthalmological clinical practice, as it offers a detailed representation of the eye fundus. This medical imaging modality is currently one of the main means of identification and characterization of intraretinal cystoid regions, a crucial task in the diagnosis of exudative macular disease or macular edema, among the main causes of blindness in developed countries. This work presents an exhaustive analysis of intensity and texture-based descriptors for its identification and classification, using a complete set of 510 texture features, three state-of-the-art feature selection strategies, and seven representative classifier strategies. The methodology validation and the analysis were performed using an image dataset of 83 OCT scans. From these images, 1609 samples were extracted from both cystoid and non-cystoid regions. The different tested configurations provided satisfactory results, reaching a mean cross-validation test accuracy of 92.69%. The most promising feature categories identified for the issue were the Gabor filters, the Histogram of Oriented Gradients (HOG), the Gray-Level Run-Length matrix (GLRL), and the Laws’ texture filters (LAWS), being consistently and considerably selected along all feature selector algorithms in the top positions of different relevance rankings.

## 1. Introduction

Nowadays, the analysis of different retinal image modalities is a crucial task as part of the diagnosis of many relevant diseases. In this context, an appropriate identification and characterization of the main retinal structures (as can be, for example, the optic disc [1] or the arterio-venular vasculature [2]) is an important issue, as the study of these structures can help to assess even diseases from other parts of our organism (as can be cardiovascular diseases [3,4] or diabetes [5]). Among the different ophthalmological image modalities, Optical Coherence Tomography (OCT) imaging reached a strong relevance, being widely used by clinicians. It offers a higher detailed representation of the retinal layer tissues, representing in a cross-sectional way the eye fundus and its structures [6]. This technique provides a more complete set of information that outperforms other classical image modalities as can be, for instance, retinographies. Moreover, their capture involve a noninvasive and contactless process that is comfortable for both patients and clinicians, making OCT a suitable technique for the analysis of relevant diseases as age-related macular degeneration (AMD) [7], epiretinal membrane [8], or macular edema [9].

**Figure 1:**
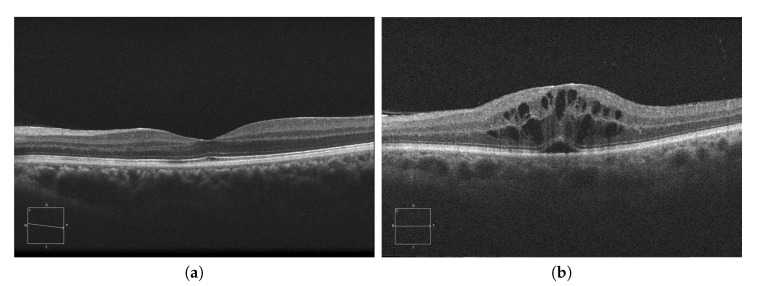
Macular Optical Coherence Tomography (OCT) images. (**a**) OCT without the presence of cystoid regions. (**b**) OCT with the presence of cystoid regions.

Currently, AMD is one of the most prevalent pathologies. It is especially relevant in developed countries, where it is among the main causes of blindness. The advance of this pathology can lead to abnormal vessel growth, resulting in fluid leakages from these malformed vessels. When this fluid begins to accumulate between the layers of the retina, the patient will begin to perceive a vision loss that may lead to blindness if left untreated. In Figure 1a, we can see an example of an OCT image of a healthy retina, whereas Figure 1b presents a retina with a perceptible accumulation of intraretinal fluid. For this reason, an early detection of these cystic bodies is critical, since it is not only an indicator of the severity of the pathology, but also helps clinicians to establish a more precise diagnosis and treatment [10].

In recent years, with the spread of the use of OCT imaging, some works have been published that face the analysis and extraction of cysts and, most of them, follow a similar strategy. These approaches frequently used an initial denoising stage to minimize the impact of the characteristic speckle noise that appears in the OCT scans. The objective is the production of clear OCT images, facilitating the posterior process of cyst detection and segmentation. Then, most of the approaches addressed directly the identification and segmentation of all the regions that are suspicious of being cysts. Finally, these works studied both shape, size and grey levels of the segmented candidates to eliminate possible False Positives (FP). With this, the aim is to correct the errors resulting from the proposed segmentation methodology, as well as to try to preserve as much as possible the true fluid accumulations.

As an example of these methodologies, we can find works like the one of Wilkins et al. [11], which proposed a methodology for the cystoid macular edema identification applying an initial thresholding to the images in order to identify dark structures as cyst candidates and a posterior use of rules to reduce the FP rates; the proposal of Roychowdhury et al. [12] also segments dark regions in bright neighborhoods in a search space defined by the 6 main retinal layers and posteriorly analyzes these identified cyst candidates in terms of solidity, mean, and maximum intensities; Wieclawek [13], that designed a method combining complementary image processing techniques to extract the cyst candidate segmentations and posterior redundant candidates removal; González et al. [14], that also produce an initial cyst candidate set segmenting dark regions using watershed and groups them by connectivity and intensity similarity (with a posterior filtering using discarding rules and a learning strategy to reduce the FPs); Esmaeili et al. [15], with K-SVD dictionary learning in the Curvelet transform to reduce the speckle noise and facilitate the application of a posterior thresholding strategy to identify the existing cysts and posterior FP removal; Wang et al. [16], which integrated the use of fuzzy C-Means and level sets in an approach called fuzzy level sets; Xu et al. [17], where the authors implemented a layer-dependent stratified sampling to produce symptomatic exudate-associated derangements segmentations using voxel classification; and Lang et al. [18], that also designed a pixel classification system, but limiting its application to the domain of microcyst segmentations.

In more recent times, deep learning techniques have also been applied, in works like the ones proposed by Lee et al. [19], Venhuizen et al. [20], Roy et al. [21], Lu et al. [22], and Chen et al. [23], which aimed too for a precise segmentation and most of them based on the U-Net architecture of Ronnenberger et al. [24], an encoder–decoder architecture with skip connections to preserve relevant features from the encoder in the decoder.

This strategy of aiming for a precise segmentation that was followed by most of the proposals presents some important drawbacks. The main limitation consists of the high dependency in the candidate segmentation stage. Poor segmentation techniques may produce significantly large candidate sets, making specially complicated the posterior FPs reduction. Therefore, large FPs sets can introduce the necessity of strong reduction processes that may carry the elimination of real identified cyst candidates. Moreover, these segmentation techniques need to be accurate, as imperfect cyst segmentations can result in FPs in the posterior segmentation refinement step.

**Figure 2:**
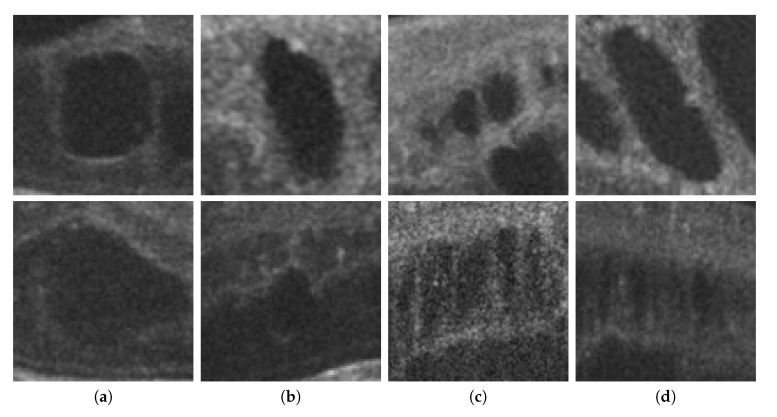
Examples with accumulations of intraretinal fluid with adequate definition for a segmentation (1st row) and counter-examples with blurred, merged, and obfuscated edges that are hard to segment precisely (2nd row). (**a**,**b**) Individual cystoid fluid bodies. (**c**,**d**) Groups of fluid accumulations.

Finally, as shown in Figure 2, 2nd row, not all cysts have a precise border that can be segmented. Many times, these fluid regions can be clearly distinguished inside the retinal layers thanks to a significant contrast between the internal fluid and the surrounding neighboring tissues (as the examples of Figure 2, 1st row show). In these cases, a segmentational approach can produce acceptable results. However, many other times this process is not as simple as the previous ones. In other cases, cysts do not present enough contrast, being extremely difficult to accurately identify their entire contour (Figure 2, 2nd row, (a,b)) or they may appear in nearby groups that make extremely complicated the identification of their limits (Figure 2, 2nd row, (c,d)). In these cases, a regional classification with the identification of the cyst presence is more feasible and also clinically relevant for the medical analysis and diagnosis.

This work presents a comprehensive study of features based on an alternative approach for the detection of these fluid accumulations. Instead of classical candidate segmentation stages and FPs removal as postprocessing, we directly identify intraretinal cystoid regions using the properties of the OCT images. To this aim, we analyzed the characteristics of the fluid cysts and the retinal layer tissues and defined a large and heterogeneous set of features for the identification of regions with and without the presence of cysts. Then, optimal subsets of features were identified using different feature selectors with the purpose of studying the suitability of the defined feature set to face this problem and the identification of those with the highest discriminative power. These subsets are posteriorly used in the training and testing of representative classifiers to evaluate the performance and behavior of the implemented methodology. The suitability of the methodology was already demonstrated in [25], showing that this approach is adequate for the analysis and identification of intraretinal cystoid regions.

## 2. Materials and Methods

**Figure 3:**
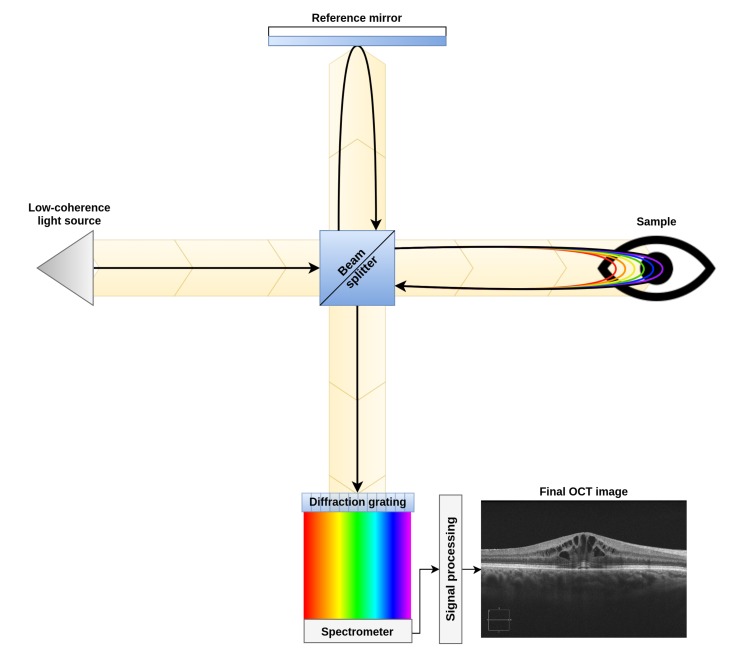
Diagram of a spectral domain optical coherence tomography device.

To perform this study, a dataset composed of 83 OCT images was created. These images were acquired from patients in vivo with a CIRRUS™HD-OCT-Carl Zeiss Meditec confocal scanning laser ophtalmoscope. This device is an Spectral Domain Optical Coherence Tomograph (SD-OCT), which uses low-coherence interferometry to study the morphology of the retinal tissues. This device, as shown in Figure 3, compares the interferences created by the tissues when the light travels through them and is reflected back into the device (as the reflectance levels of different tissues will cause different interference patterns in the returned signal). This particular device uses and spectrogram and the Inverse Fast Fourier Transform (IFFT) in the signal acquisition step. This allows to study, in one single take, different tissue depths by analyzing different ranges of frequencies detected in the spectrogram. This signal acquisition technique allows to recover all the points of an A-Scan (each of the columns of an OCT image) at the same time. Finally, by merging all the A-Scans into a single B-Scan, the device generates the final OCT image [26,27].

A large variability is represented in the dataset, including images from 750×500 pixels to 1680×1050 pixels. These images were taken centered in the macula from both eyes of different patients and multiple device configurations. Also, no preprocessing techniques were used, aiming to preserve the original characteristics of the eye fundus.

The image dataset was labeled by an expert clinician, identifying all the cysts that appear in the images. Using this ground truth as reference a sample dataset was built, including 806 and 803 windows with and without the presence of cysts, respectively, summing a total of 1609 samples. For dataset creation, sample extraction and labeling, and feature vector extraction we have used the MATLAB R2018a suite. On the other hand, for the training of classifiers, result analysis, and the creation of the different graphs of this work, we have used the Pandas [28], Seaborn [29], Scikit-learn [30], Matplotlib [29], and Numpy [31] libraries for Python 2.7.

**Figure 4:**
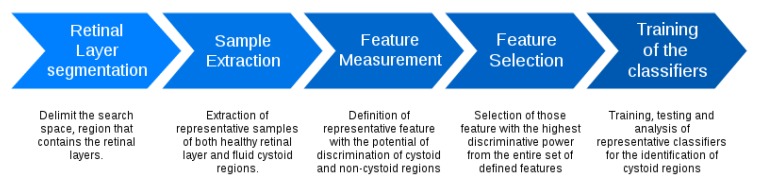
Main stages of the used methodology.

Figure 4 presents the main stages of the used methodology. The system firstly identifies the retinal layers that delimit the region of interest. Then, overlapping square windows are analyzed in order to determine the presence of containing cysts. Then, each analyzed window is represented by a complete set of features that are posteriorly studied to determine those optimal subsets that maximize the cyst identification. Finally, the method includes the analysis of representative classifiers to determine the performance of the proposed system. Each stage is detailed in the following subsections.

### 2.1. Retinal Layer Segmentation

The region of interest (that is, the area where the fluid accumulations can appear) is between Inner Limiting Membrane (ILM) and the Retinal Pigment Epithelium (RPE). The ILM is the first retinal layer, and the RPE is formed by pigmented cells at the external part of the retina.

**Figure 5:**
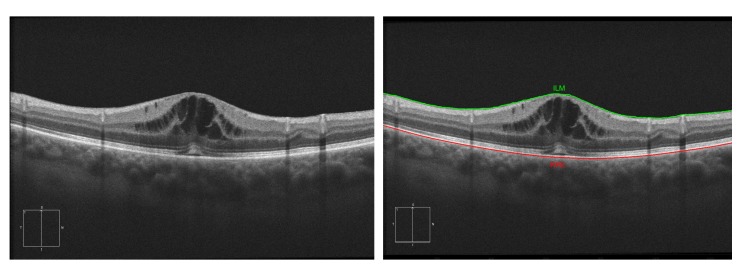
Example of the segmentation of the Inner Limiting Membrane (ILM) and Retinal Pigment Epithelium (RPE) retinal layers.

For this stage, we based our approach on the work of Chiu et al. [32]. This algorithm considers the pixels of the image as a graph of nodes, being the vertical dark-to-light gradients the weights of the path. These vertical gradients, when present, usually define the limits of a retinal layer. Using the algorithm of Dijkstra [33], we find the minimum paths from one side of the OCT image to the other. Then, using this path as reference, we delimit the search area for other secondary paths (such as the other retinal layers). Despite this methodology being able to segment all eight main layers, for this work, we only identified the ILM and RPE (as all the cysts will be contained between these two layers). Figure 5 presents an illustrative example of the ILM and RPE retinal layer identification in an OCT image.

### 2.2. Sample Extraction and Feature Measurement

Over the identified region of interest that is delimited by the retinal layers, the system analyzes square windows of a defined size to determine the presence or non presence of cysts. Therefore, each analyzed window is characterized by a large and heterogeneous set of 510 features, including mainly intensity and texture-based features. This way, we try to capture, as much as possible, the main properties of the cystoid regions so that they may be distinguished from the typical normal retinal layer tissue or other patterns that may appear in the retinal layers of the OCT scans. Table 1 details the entire list of features that were considered in this work.

#### 2.2.1. Global Intensity-Based Features (GIBS) and Axis Intensity Statistics (AIS)

Typically, cystoid regions imply a higher irregularity and a drop in the intensity profiles with respect to the normal tissue of the retinal layers. For that reason, we measured global statistics of the analyzed region with the purpose of capturing these alterations. In particular, we have maximum, minimum, mean, median, standard deviation, variance, entropy, 25th and 75th percentile and maximum likelihood estimates for a normal distribution. On the other hand, the mean, minimum and maximum skewness, and kurtosis along the vertical and the horizontal axis were calculated to further study the cyst distribution in the sample. Patterns like shadows and healthy retinal tissue should present similar levels along at least one axis, whereas fluid accumulations would tend to have more heterogeneous values in both axis due the tendency to form spherical and fusiform structures.

#### 2.2.2. Eigenvalues

In this work, the use of eigenvalues can provide information about samples with intensity changes if a wide range of directions, such as when hyporeflective fluid bodies are present surrounded (or partially covered) by normal retinal tissues. We selected the four highest ($\lambda_{max_{i}}$) and the four lowest ($\lambda_{min_{i}}$) eigenvalues. Additionally, several ratios among them were also included in the feature set, as well as the 25 and 75 percentile. If the maximum rectangular ROI area inside a sample (region where the features are actually extracted) is not square, a sliding window strategy inside this rectangular ROI is used to calculate the eigenvalues.

#### 2.2.3. Local Energy-Based Shape Histogram (LESH)

Proposed by Sarfraz et al. [34], Local Energy-Based Shape Histogram (LESH) features analyze the texture energy using Gabor to obtain the magnitude and phase of the pixels in the sample. They study the points of maximal phase congruency [35]. This feature descriptor uses Kovesi’s [36,37] phase congruency approximation as energy measure, making it robust to noise. Its response highlights edges and borders, making it useful to detect shadows, retinal layers, cyst membranes, and other high contrast non-homogeneous elements that may be present in the region of interest. This feature descriptor has proven its usefulness in other medical imaging domains, being successfully used to detect breast [38] and lung [39] cancer. These cases also present tissues with an heterogeneity difference between healthy and malignant regions.

#### 2.2.4. Gray-Level Co-Occurrence Matrix (GLCM)

Other texture-based features that may be useful to face this issue are the GLCM features. These second-order statistics measure the simultaneous occurrence of gray levels *i* and *j* in pairs of pixels ($p_{1},p_{2}$) of the analyzed OCT image *I*, separated by a displacement vector δ = (Δx, Δy) into a 2D matrix, given by

(1) $$C_{\delta }\left(i,j\right)=\left|\left\{p_{1},p_{2}:I\left(p_{1}\right)=i;I\left(p_{2}\right)=j;p_{2}=p_{1}\pm \delta \right\}\right|$$

Based on the proposal of Haralick et al. [40], different texture features may be extracted from the co-occurrence matrices. The experiments were performed using a distance of 2 pixels in the following angles; 0${}^{\circ }$, 45${}^{\circ }$, 90${}^{\circ }$, and 135${}^{\circ }$. Thus, we obtain a total of 16 markers for this feature class.

#### 2.2.5. Histogram of Oriented Gradients (HOG)

The orientation of the gradients that may appear in the analyzed regions is a key property to identify the hypothetical presence of cysts. This is derived from the fact that cysts typically present closed/oval contours, producing a significant number of gradients and a large variability in their orientations. On the contrary, non-cystoid regions normally present a more uniform shape, with a lower level of gradients. Also, when they are present, they typically present parallel horizontal patterns (introduced by the presence of the retinal layers) or vertical and tubular patterns (introduced by the shadows of vessels or other structures that appear in the upper parts of the retinal layers). HOG features [41] are suitable to capture these different patterns from cystoid and non-cystoid regions. They also present some invariance to scale, rotation or translation changes, properties that are useful in this issue given the significant variability in terms of size, shape, and locations of the cysts inside the retinal layers. Nine HOG windows per bound box and nine histogram bins were considered in this work, summing a total of 81 analyzed HOG features.

#### 2.2.6. Gabor Filters

Other popular texture-based features, widely used in medical imaging solutions, are Gabor filters [42]. One of the main advantages of Gabor filters, with the proper configuration, is their invariance to translation or scale transforms, being suitable for the cyst identification issue of this work. Additionally, Gabor filters also present a remarkable robustness to deformations in the image as intensity variations or significant levels of noise, common conditions that are normally faced in the analysis of OCT scans. Using the OCT image as reference, two-dimensional Gabor filters are calculated [43] as a Gaussian kernel function modulated by a sinusoidal wave represented by

(2) $$G\left(x,y\right)=\frac{f^{2}}{\pi \gamma \eta }exp\left(-\frac{x^{{}^{\prime }2}+\gamma^{2}y^{{}^{\prime }2}}{2\sigma^{2}}\right)exp\left(j2\pi fx^{\prime }+\phi \right)$$

where *f* represents the frequency of the sine wave, θ the orientation, σ indicates the standard deviation, γ is the aspect ratio (which measures the ellipsoidal elongation of the filter in the direction of the normal of the orientation), ϕ is the phase offset, and η the sharpness along the minor axis, where $x^{\prime }$ and $y^{\prime }$ are represented by

(3) $$\begin{array}{c} x^{\prime }=x\cos\theta +y\sin\theta \\ y^{\prime }=-x\sin\theta +y\cos\theta \end{array}$$

In this work, a bank of filters was defined using eight scales of frequencies, *f* in combination with the same number of orientations θ [44]. Finally, global statistics as *mean* and *standard deviation* are finally calculated using the generated feature vectors to obtain a total of 128 Gabor features.

#### 2.2.7. Local Binary Patterns (LBP)

Texture-based features can be very useful to capture the patterns that appear with the normal retinal layer tissues as well as the irregularities that are produced by the cyst presence or the fluid tissue that is contained inside the cysts. In that line, LBPs texture-based features [45] are frequently used in the design of medical imaging systems. Moreover, LBPs present low sensitivity to changes in intensity. This is an advantage, as different OCT devices generate images with different brightness, noise, and contrast conditions. Moreover, even considering the same device, the use of different configurations and the time needed for the image acquisition significantly affects this factor. For this reason, the fact that LBP is unaffected by changes in the overall image intensity gives it additional robustness not present in several of the other features classes. Also, a rotation-variant version is used in this work, as both rotation-variant and rotation-invariant versions were previously tested and the former obtained slightly better results (as texture orientation proved to be a relevant factor in this problem). A wide range of LBPs were analyzed, using filter sizes of 4, 8, 12, and 16 and radius from 1 to 8. Finally, from the obtained descriptors *mean* and *standard deviation* are calculated summing a total of 64 LBP features.

#### 2.2.8. Laws’ Texture Filters

Developed by Laws [46], this descriptor consists of a series of 2D convolution kernels focusing on different texture properties. These kernels are usually generated from a set of one dimensional kernels. In this work, we considered the following kernels for window of sizes 3×3 and 5×5; L3 = [1 2 1], E3 = [−1 0 1], S3 = [−1 2 −1], L5 = [1 4 6 4 1], E5 = [−1 −2 0 2 1], S5 = [−1 0 2 0 −1], and R5 = [1 −4 6 −4 1]. L kernels focus on obtaining the center weighted gray level mean, E kernels respond to edge features, S extract spots, and R focus on ripple patterns in the texture.

#### 2.2.9. Fractal Dimension (FD)

This filter first transforms the image from grayscale into the FD domain [44]. This allows us to study the fractal properties of the different image textures. These features are useful to capture texture information that, otherwise, would be hidden in normal intensity patterns. The fractal transformation of the window is performed using a box-counting (DBC) algorithm [47]. Using this fractal transformed image, different general statistics can be calculated. In our case, we derive *mean*, *standard deviation*, and *lacunarity*, which measures the texture rugosity. This texture descriptor can be useful to detect complex cystoid regions difficult to segment, as they are a mixture of both fluid and retinal tissue presenting a rough texture pattern.

#### 2.2.10. Gray Level Run Length Image Statistics (GLRL)

Consisting of a consecutive, collinear picture points having the same gray level, Loh et al. [48] proposed to measure the length and properties of indicated runs to obtain the size of the texture elements. If this idea is extended to multiple rotations, the shape and texture alignment is also obtained through this texture descriptor. To diminish the effect of the image noise and slight luminosity variations, the total possible 256 gray levels were grouped into 51. With the resulting matrix, the Run Percentage (RP), Run Length Nonuniformity (RLN), Low Gray Level Run Emphasis (LGRE), High Gray Level Run Emphasis (HGRE), Short Run Emphasis (SRE), Long Run Emphasis (LRE), and Gray Level Nonuniformity (GLN) were measured.

This texture descriptor is particularly interesting, as cystoid fluid areas are represented by nearly homogeneous low gray level zones while healthy tissues tend to have more heterogeneity. Given the multiple orientation nature (obtaining thus the cyst shape), similar bodies that could be confused with cysts can also be easily identified. An example of these cases can be shadows, which are vertical rectangular areas caused by the OCT light being distorted by a dense body in more internal layers. Other pathological non-fluid hyperreflective homogeneous structures like drusen or exudates can also be detected by this descriptor. As some of these structures tend to appear together with cystoid bodies, the system can take advantage of them to find a correlation between these two patterns and help to further tune the results.

### 2.3. Feature Selection

Feature selection is an important stage of any computer-aided diagnosis system, especially when the defined feature sets are significantly large, as happens in this work with the definition of a large set of 510 features. In such cases, we can assume the existence of redundant or irrelevant features and reduce the feature dimensionality to a optimal subset, keeping the ones that best characterize the target classification without compromising the class separability and optimizing the system in terms of computational costs.

The objective of a selection strategy is the identification of those features that provide a large distance between the classes as well as a small variance within the classes. There are numerous algorithms that perform the selection process. In this work, three representative feature selector strategies were used: Trace ratio estimator [49,50], Relief-F [50,51,52], and a ensemble-learning based random forest (RF) feature importance estimation using the extra randomized trees variation [30,53]. The trace ratio approach by Nie et al. [49] finds the subset of features for which the score is maximized, thus finding the features where the within-class affinity is the lowest and highest between samples from different classes (being the affinity represented by the weights of the undirected graphs for both relationship types). Relief-F samples instances randomly and verifies the distance between them and their neighbors that have the same or different classes. A weight vector is created using the distances to rank them. Finally, the ensemble method trains a RF classifier and then evaluates the depth of each feature in the tree (as features used at the top of the tree contribute more to the final prediction) to asses their relative importance.

### 2.4. Classification

Finally, using the selected feature sets, we evaluate the performance of representative classifiers. In particular, we tested a *Linear Discriminant Analysis* (LDA), *Ridge-regularized linear model*, two *Support Vector Machine* (SVM) configurations, a *k Nearest-Neighbors*(kNN) with k=15, a RF classifier, and a *Gradient Boosting* (GB) ensemble classifier [30].

The LDA uses Bayes’ rule to fit a Gaussian density to each class. The model assumes that all classes share the same covariance matrix, thus employing singular value decomposition to optimize the between class scatter-within class scatter ratio.

The kNN classifier [54] is a classifier that estimates the probability of belonging to a class based on the number and distance to the training dataset samples belonging to that class. In this case, the number of neighbors *k* is the hyperparameter to optimize. In this work, we used as reference k=15 as the one that offered an accurate performance. Additionally, for each query, the points are weighted by the inverse of their distance, making closer points more influential when determining the resulting class.

The SVM classifier [55] separates two classes by looking for the hyperplane that maximizes the distance between the two closest samples of two classes (called support vectors). In principle, SVMs converge for linearly separable data. However, for more complex boundaries, we can use the “kernel trick”. This involves an implicit transformation of characteristics to a space where a linear separation is possible. Two configurations were considered in this case: a linear SVM (no kernel) and a RBF (Gaussian) kernel.

As an ensemble method, RF [56] consists of a forest of randomized trees built with bootstrap sampling from the training set. This algorithm creates a diverse set of classifiers by introducing randomness in the classifier construction [57]. The classifiers are combined by averaging their probabilistic prediction, and the node splitting is made by picking the best split among a random subset of features.

The GB classifier [58] is another ensemble classifier that sequentially builds an additive model. A total of 100 fixed size regression trees are trained and the log-likelihood optimized in each iteration via gradient descent towards the negative gradient of the loss function.

Finally, the Ridge classifier trains a linear regression model for classification, but imposing an additional constraint to the loss function to prevent overfitting. Ridge imposes a penalty on the size of the linear regression coefficients, minimizing a penalized residual sum of squares. Ridge belongs to a family of classifiers that use regularization to prevent overfitting by reducing/simplifying the resulting model. Commonly, L1 and L2 penalties are used, Ridge basing itself on a L2 penalty. In this work, we preferred this L2 regularization over an L1 because this last one may remove features by turning its coefficients to zero. We want to analyze the response of the classifier to all the features in the given subset and not perform an underlying secondary feature selection. Thus, an L2 penalty is more appropriate for this study.

The classification was done using a constructed dataset that was randomly divided into a 10 fold cross-validation repeated 10 times for each selector and classifier configuration. The feature selection was performed using only the train set to prevent a possible bias in the test results, and a model was trained for each increasing subset of features to further study the behavior of each selected feature to a max of the top 100 selected features. This limit was established empirically, as no further significant improvement in the results was shown. By doing so, we end with 700 top 100 feature rankings for each selector (100 rankings per classifier) and 30,000 accuracy values for each classifier (100 test cross-validation values × 100 feature subsets per ranking × 3 feature selection strategies).

## 3. Results

**Figure 6:**
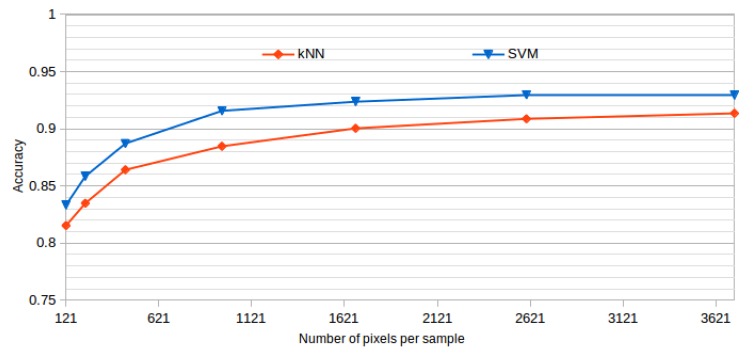
Window size analysis. Best accuracy obtained by each tested classifier and progressive larger window sizes.

All the presented results were obtained using a window size of 61 × 61, as it offered the best results while also representing a good balance between the inclusion of information and the detection details. This size was established after analyzing the performance of the system using progressive window sizes in a preliminary test with representative kNN and SVM classifiers and a reduced number of features. In the dataset construction, we built the corresponding datasets using the same central points in all the cases, but using progressive window sizes. Table 2 and Figure 6 summarize the mean test accuracy results that were obtained using each window size.

As shown, the performance of the methodology is increasing over higher window sizes, as bigger windows provide more information to capture the cystoid and non-cystoid patterns. We can see that at a window size of 61 × 61, the accuracy is already stabilized. Selecting bigger windows would penalize the computational costs without providing significant improvements.

**Figure 7:**
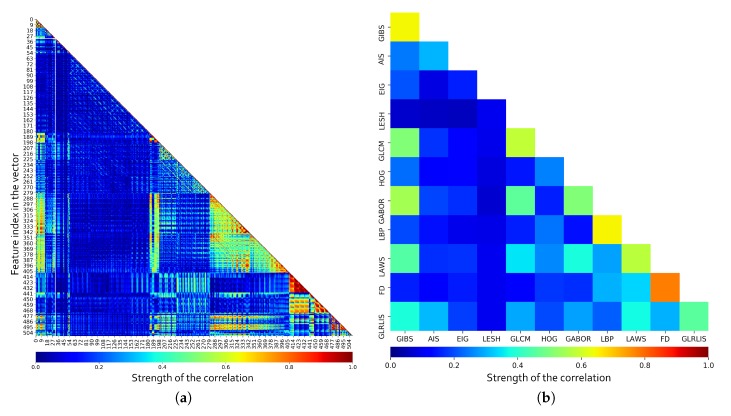
Pairwise correlation for each individual marker in the feature vector (**a**) and mean correlation by feature class (**b**).

To evaluate the features, first of all, the pairwise correlation between all the features was calculated. As shown in Figure 7a, we have some features offering partially or totally redundant information even with markers from the same category. For a better understanding of the values, the mean correlation per feature category is included in Figure 7b. Furthermore, the correlation values between the same markers were ignored when calculating the mean correlation, so the results also present information about the intraclass correlation. Note how, as overall, some classes present low correlation values (like the LESH descriptors or Eigenvalues), but also how others contribute with similar information at some level as other descriptors (like Gabor with GIBS and GLCM). Also, some categories like FD, LBP, and GLCM present a high level of intraclass correlation, indicating that a subset of the descriptors from that same category can cover mostly of the information provided by the entire set. These test reveal the need to perform a feature selection in order to remove non-relevant and redundant information while reducing the computational cost. Being so, we analyze several feature selection strategies and classifiers to determine the relevance of the described features.

To evaluate these features, a 10-fold cross-validation repeated 10 times was performed for each classifier and feature selection strategy. The most relevant features are calculated for each training fold set, preventing the results of the classification to be influenced by the additional information the feature selector may add from the test subset. Finally, for each fold, the tested classifier is trained using progressive larger feature subsets.

**Figure 8:**
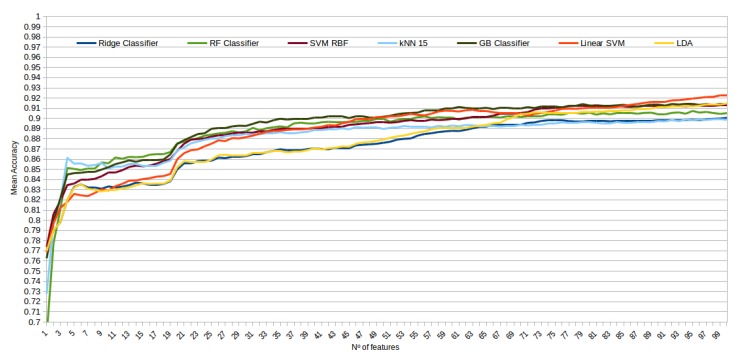
Evolution of the accuracy for each classifier tested with an increasing subset of features obtained with the trace ratio selector.

**Figure 9:**
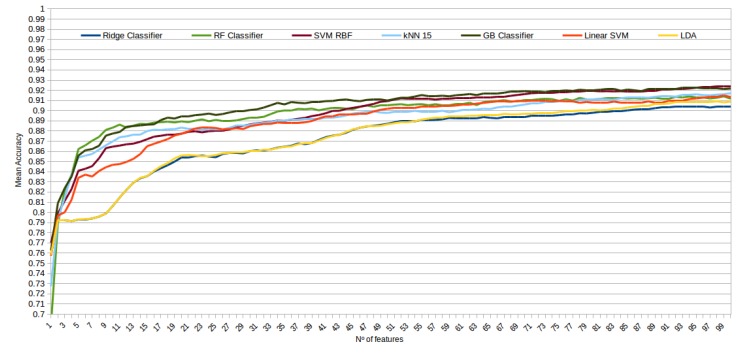
Evolution of the accuracy for each classifier tested with an increasing subset of features obtained with the random forest (RF) ensemble selector.

**Figure 10:**
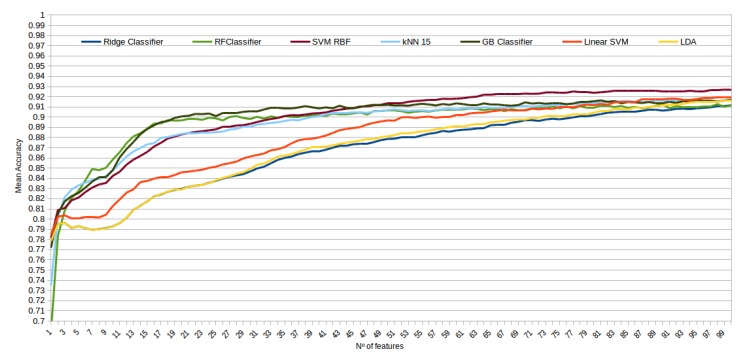
Evolution of the accuracy for each classifier tested with an increasing subset of features obtained with the trace Relief-F.

The mean accuracy that was obtained for all the folds and repetitions can be seen in Figure 8 using the trace ratio feature selector, Figure 9 using the RF ensemble selector, and Figure 10 using the Relief-F selector, respectively. A maximum size of 100 features was established, as no significant improvements were obtained with bigger extracted feature subsets. Adding more features would only diminish the model generalization capabilities. Generally, we can observe how the forest-like classifiers (RF and GB) tend to provide better performances with less features, but with larger feature subsets the SVM with RBF kernel attains better results. Moreover, the trace ratio selected features are the only ones who presenting a similar pattern between linear and nonlinear classifiers, while Relief-F and Random Forest selected features clearly are favorable to nonlinear classifiers with smaller feature subsets.

**Figure 11:**
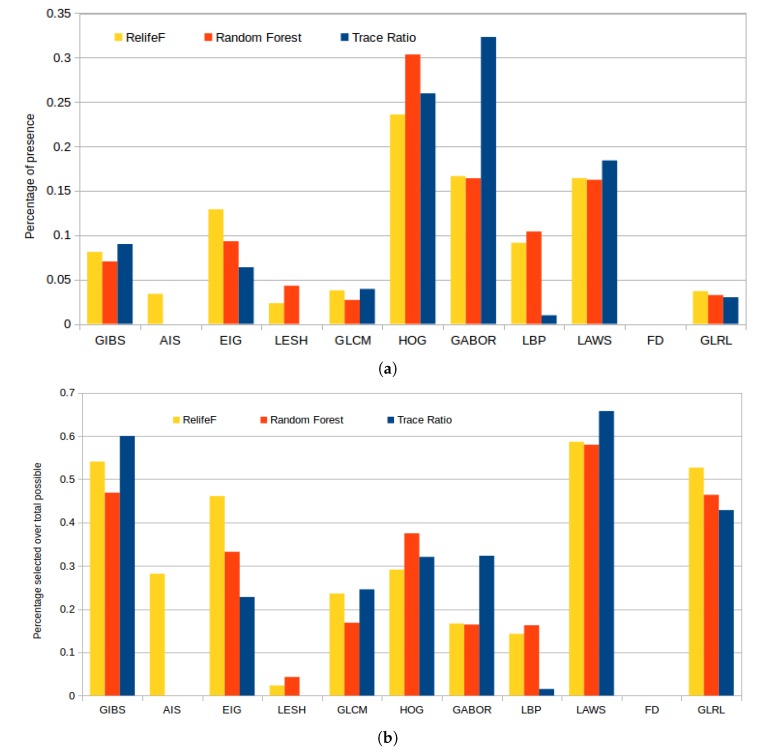
Percentage of presence of each family of features in the top 100 of each feature selector (**a**) and proportional percentage of selection relative to the maximum possible number of features for each family of features (**b**).

Figure 11a represents the selected features by each strategy, of a total of 100, grouped by the corresponding feature categories. In absolute terms, we can see that HOG features were largely included by all the strategies because, as indicated before, they accurately capture the large variability of the gradient orientations that are derived from the presence of cysts or the uniform gradient patters derived from the normal retinal tissues. Gabor and LAWS features were also selected similarly along the different folds, as there is a level of correlation between them (the ripple texture patterns and the center-weighted image mean can be captured with both descriptors). Finally, we can see how Relief-F greatly favored Gabor and considerably reduced the number of LBP features included in its subset, but behaved similarly with the rest of the feature categories.

Given the large disparity in the number of markers per feature class, we also present in Figure 11b statistics independent of the feature category size. These consists in the percentage of presence in the top 100 considered features by each selector over the maximum possible (that is, 100 or the maximum available number of markers for any given class). This reveals how, despite the apparent low selection rate in Figure 11a of GIBS, LAWS, or GLRL, it is shown that almost all the features from these categories were selected. We have to take into account that these features do not have a number of descriptors for selection as high as Gabor or HOG so, despite their low values in the previous bar plot, these were also significantly chosen and have to be considered in further analyses.

**Figure 12:**
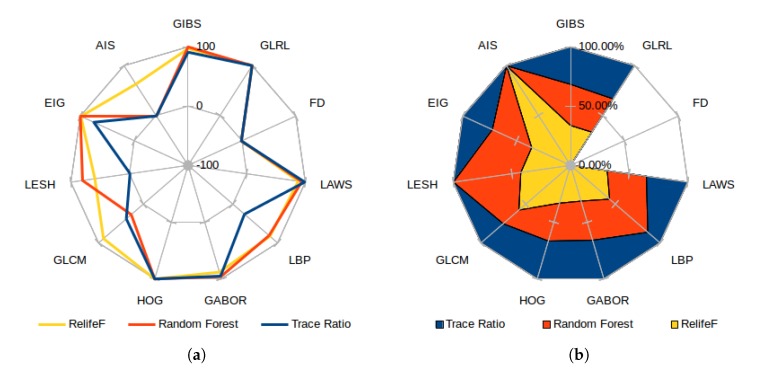
Best score attained for each feature category (**a**) and proportional feature relevance over maximum achieved position (**b**) for each feature selector.

As the selectors use the feature positions in the ranking to express their relevance, Figure 12a presents the score for each category, measured as the inverse of the minimum achieved index in all the folds (or −1 if the feature category has no presence whatsoever in the top 100 considered features). This means that a feature category with a score of 100 has a marker chosen as the most important feature. A score of 0, on the other hand, represents the less relevant feature of the top 100. Both graphs reflect the already commented preference of most selectors for GLRL, LAWS, GABOR, and HOG (closely followed by the Eigenvalues and GIBS features), moving around the top position along the folds and obtaining a similar proportional relevance by all the analyzed selectors. In Figure 12b, which presents the proportional relevance given by each selector over the maximum score attained, note how all the features deemed relevant offer similar proportional weight in all the considered selectors; while the most controversial are favored by ones and greatly disregarded by others. Also, Relief-F was the selector which offered a higher heterogeneity when adding new features to the top, reflected as an overall minimum high index over the selected feature classes. On the other hand, the trace ratio selector only favored the candidates mostly chosen by all the classifiers, being the only one that ignored classes as LESH or AIS, selected by at least one of the other selectors.

**Figure 13:**
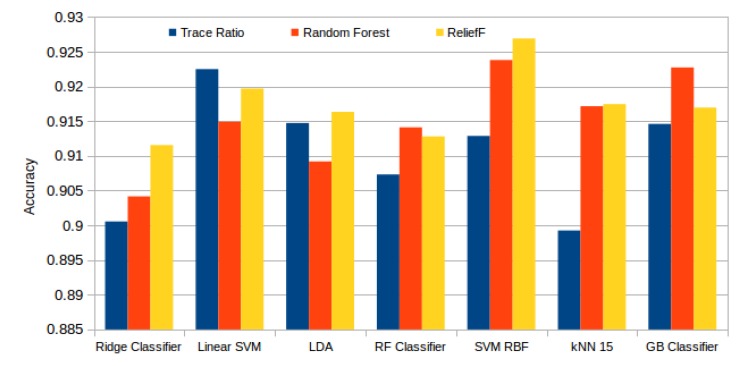
Best mean accuracy achieved by each classifier and selector.

Figure 13 presents the best results attained by each classifier and selector. Here we can see how Relief-F achieved generally the best results, surpassed by the Random Forest selector only with the ensemble tree based models (Random Forest and Gradient Boosting). In general terms, the performance that was reached with each configuration is satisfactory. The best mean accuracy was achieved by the SVM RBF model with the subset of the 99 best features selected by the Relief-F algorithm. Despite this high number of features needed, and as seen in Table 3, the last features have a low impact in the final result as the system has already stabilized. The last 50 features have a mean difference between the accuracy of the immediate previous subset of 0.00029, with an standard deviation of 0.00054.

## 4. Discussion

The tests presented in the previous section indicate a clear relevancy of features from GABOR, HOG, LAWS, and GLRL categories, having a considerable presence in the rankings while also maintaining consistency along all three distinct feature selection strategies. GIBS and Eigenvalues were also highly chosen in the top features, but with a slight discrepancy between the feature selection strategies. Note that the FD features were the only completely omitted by all selectors, probably due to the capabilities of the other feature descriptors to fill his gap while also offering additional information.

All these relevant feature descriptors have in common the study of gradient orientations and gray level intensities in the samples. Fluid accumulations usually present an homogeneous dark texture compared to the other retinal structures. This is caused because these fluids do not interfere with the coherent light beam from the device as much as normal retinal tissues, presenting so an hyporeflective homogeneous pattern. Regarding gradient orientations, normal structures in the retina present horizontal or vertical patterns. Horizontal patterns are common because of the morphology of the retinal layers, and vertical patterns usually occur when a shadow is projected by a dense body in a superior (more internal) layer. On the other hand, as cystoid structures usually adopt a spherical shape, they present gradients in all the possible orientations. Even if the cystoid area is considerable compared to the established window size, several of the gradient orientations will not be present anywhere else in the OCT image. Additionally, these unique gradient orientations also occur in regions where the fluid appears to be mixed with normal tissue, further explaining the reason of the unanimous disregard for the FD features (as the other descriptors already help in the cases covered by them).

Finally, despite HOG features being the only descriptor favored by the selectors that does not study the intensity, it offers consistent results independently on the contrast and gray level distribution of the sample. This can be helpful in situations where the normal retinal tissues present similar dark patterns as the fluid regions but the borders are still present (thanks to their slightly higher density). This density variation in the OCT image that delimits the cystoid region is caused by the membrane formed in these fluid accumulations, when they attain certain size.

**Figure 14:**
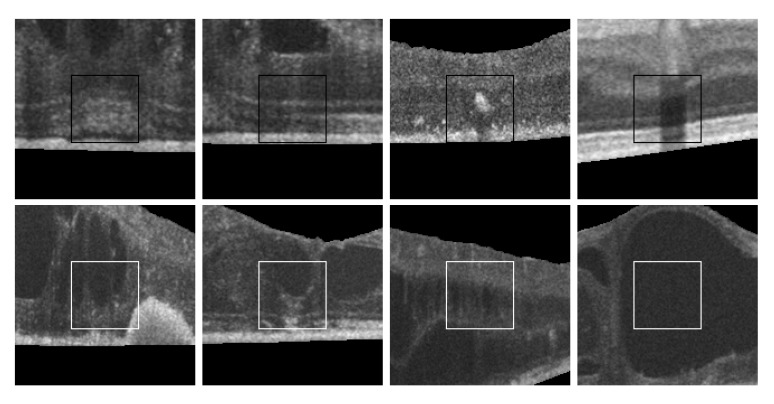
Randomly selected samples from the test dataset labeled by experts that were correctly classified by the automatic system. 1st row, non-cystoid regions. 2nd row, cystoid regions.

Figure 14 includes some representative cystoid and non-cystoid regions that were selected from a test dataset and that were correctly classified. In the case of non-cystoid regions, the method is not only capable to identify the typical retinal layer patterns, but also other regions with a large diversity of irregularities. Thus, Figure 14, 1st row, shows some examples with the presence of pathological alterations or the projection of shadows that alter the intensity patterns. Generally, these cases were correctly handled by the method. Some representative cystoid regions are also presented in Figure 14, 2nd row, with examples of different levels of complexity including cysts with irregular contours, mixed with other pathological structures, nearby groups of cysts or fluid regions inside huge cysts, respectively, cases that the system was capable to handle.

**Figure 15:**
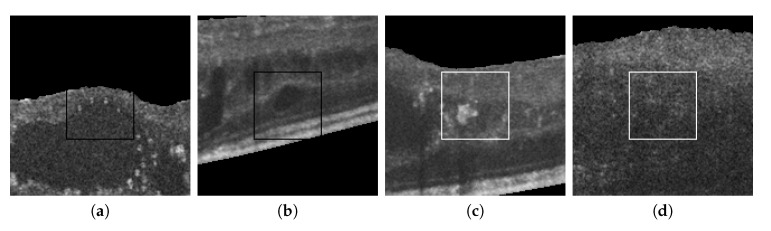
Randomly selected samples from the test dataset labeled by experts that were incorrectly classified by the automatic system. (**a**,**b**) Cystoid regions classified as non-cystoid. (**c**,**d**) Non-cystoid regions classified as cystoid.

Figure 15 also presents some incorrect classifications. Many misclassified cystoid regions are originated by the poor contrast and fuzzy contours that sometimes are present in the OCT scans. Other times, the presence of other pathological structures can result in FPs, creating artificial high-to-low intensity regions similar to the patterns present in real cystoid regions.

## 5. Conclusions

An appropriate identification and posterior analysis of any existing intraretinal cystoid fluid region is crucial for the early diagnosis and treatment of diseases like the exudative macular disease or macular edema, pathologies that are included among the main causes of blindness in developed countries. To date, most of the existing methodologies faced this issue following, generally, the same strategy: image preprocessing, an initial segmentation of all cyst candidates, candidates are posteriorly analyzed in terms of intensity and morphological properties to reduce the FP rates, and preserve the final cyst identifications. This strategy includes some important drawbacks. It presents a strong dependency in the accuracy and robustness of the candidate segmentation phase, given the complexity of the OCT images and the difficulty of segmentation of many cysts. In these cases, the method can omit or segment imperfectly the aimed existing cysts. Moreover, the candidate segmentation phase can produce large candidate sets, hardening the posterior FP removal. Therefore, imperfect or wrong segmentations as well as huge candidate sets can lead, frequently, to remove real existing cysts or preserve FPs in the posterior intensity/morphological analysis which, therefore, penalizes the performance of any proposed system.

This work uses a robust methodology for the automatic identification of intraretinal cystoid fluid regions using OCT scans to perform a complete and comprehensive study of the relevant features that better describe these fluid accumulations. Instead of the previously indicated strategy of candidate segmentation and FP reduction, this methodology faced this issue with a different strategy. We performed an analysis by regions inside the retinal layers and determined the presence or non-presence of containing cysts. This way, this strategy overcomes the drawback of the segmentation stage directly detecting the cystoid regions inside the analyzed OCT images.

The methodology includes a complete analysis of the features that help in the cystoid fluid identification. For this purpose, 510 intensity- and texture-based features were implemented and extracted over each analyzed region. The inclusion of these features was made studying the main characteristics of the typical cystoid presence or absence. In order to remove redundant and irrelevant information, 3 different feature selection strategies were applied to the extracted feature sets. In particular, we tested the trace ratio maximization strategy, the Relief-F algorithm and an ensemble random forest classifier as a feature selector to obtain more global and consolidated conclusions about the performance of all types of features. Then, different representative classifiers as a Ridge-regularized classifier, Random Forest, different SVM configurations, a kNN, a Gradient Tree Boosting, and a LDA were trained and tested using the image datasets and the selected features with the aim of the obtaining of the best combination of feature selection set and classifier.

The proposed method was validated using a set of 83 images. From these images, 806 cystoid and 803 non-cystoid samples were extracted, summing a total of 1609 samples. Considering aspects like the number of features whose accuracy variation influence was deemed relevant for each category, the stability of the selection across the multiple feature selection algorithms and the position these features fell on their respective rankings; GABOR, HOG, LAWS, and GLRL were greatly favored over the other categories. The intensity based features (GIBS) and Eigenvalues were the second most important identified group to be considered relevant. Regarding their behavior with the classifiers, all the tested configurations achieved satisfactory results, reaching a mean best accuracy of 0.9269 with the SVM classifier with an RBF kernel. This accuracy was attained using a subset of 99 features provided by the Relief-F feature selector and a window size of 61×61 pixels. Despite the high number of features needed to achieve the aforementioned result, it has been proven that the accuracy improvement with more than 50 features is negligible as the system has already stabilized.

As we could see in the results, the system is accurate in the cyst identification, but some problems still remain specially with microcysts. Gabor filters, for instance, demonstrated to be very useful detecting the inner fluids of the cysts, but they present a reduced performance when dealing with cysts of lesser sizes and, consequently, tiny fluid areas. We could design specific classifiers for each situation including a trained classifier for these specific microcyst cases that would be combined with the accurate performance of the previous trained classifier. This microcyst learning case could also adapt the used features or the window size and complement the weakness of the general process.

## Figures and Tables

**Table 1:** Measured features. *max* - Maximum; *min* - Minimum; *sd* - Standard deviation.

Category	Features
Global Intensity-Based Features (GIBS)	[1–9] Max., min., mean, median, sd., variance, entropy and percentiles 25 and 75. [10–15] Maximum Likelihood Estimates.
Axis Intensity Statistics (AIS)	[16–19] Min. skewness and kurtosis. [20–23] Mean skewness and kurtosis. [24–27] Max. skewness and kurtosis.
Eigenvalues	[28–55] Top, bottom eigenvalues and different relations between them.
Local Energy-Based Shape Histogram	[56–183] Descriptors using 5 scales, 8 orientations and 16 partitions.
Gray-Level Co-Occurrence Matrix	Distance of 2 pixels and 4 directions: 0${}^{\circ }$, 45${}^{\circ }$, 90${}^{\circ }$ and 135${}^{\circ }$. [184–187] Contrast [188–191] Correlation [192–195] Energy [196–199] Homogeneity
Histogram of Oriented Gradients (HOG)	[200–280] 9 windows and 9 bins per window.
Gabor filters	8 orientations and 8 scales [281–312] Mean [313–408] Standard deviation
Local Binary Patterns (LBP)	Number of neighbors: 4, 8, 12 and 16 pixels. Filter radii: 1 to 8 pixels. [409–440] Mean [441–472] Standard deviation
Laws’ texture filters	[473–486] Mean [487–500] Standard deviation
Fractal dimension features	[501–503] Mean, std. and lacunarity.
Gray Level Run Length image statistics (GLRL)	[504] Short Run Emphasis (SRE) [505] Long Run Emphasis (LRE) [506] Gray Level Non-uniformity (GLN) [507] Run Percentage (RP) [508] Run Length Non-uniformity (RLN) [509] Low Gray Level Run Emphasis (LGRE) [510] High Gray Level Run Emphasis (HGRE)

**Table 2:** Window size analysis. Best accuracy obtained by each tested classifier and progressive larger window sizes.

*Window Size*	*11 × 11*	*15 × 15*	*21 × 21*	*31 × 31*	*41 × 41*	*51 × 51*	*61 × 61*
*Pixels per window*	*121*	*225*	*441*	*961*	*1681*	*2601*	*3721*
**kNN**	0.8153	0.8348	0.8641	0.8847	0.9004	0.9087	0.9135
**SVM**	0.8333	0.8584	0.8871	0.9157	0.9237	0.9297	0.9294

**Table 3:** Mean accuracies attained by the best selector for each classifier. The granularity of the table reduces as the feature subset size increases because of the lower accuracy variation between subsets.

N. Features	1	5	10	20	30	50	70	100
*Ridge Classifier*	0.7785	0.7932	0.7929	0.8296	0.8467	0.8786	0.8968	0.9116
*Random Forest*	0.6890	0.8623	0.8833	0.8895	0.8930	0.9053	0.9103	0.9118
*RBF SVM*	0.7811	0.8211	0.8424	0.8830	0.8934	0.9135	0.9231	0.9268
*kNN 15*	0.7348	0.8331	0.8474	0.8836	0.8908	0.9069	0.9110	0.9175
*Gradient Boosting*	0.7718	0.8255	0.8482	0.9007	0.9057	0.8120	0.8145	0.9170
*Linear SVM*	0.7688	0.8259	0.8292	0.8599	0.8816	0.9011	0.9045	0.9225
*LDA*	0.7785	0.7933	0.7928	0.8292	0.8493	0.8811	0.8979	0.9163

## Abbreviations

The following abbreviations are used in this manuscript:

OCT	Optical Coherence Tomography
HOG	Histogram of Oriented Gradients
GLRL	Gray-level Run-length matrix
LAWS	Law’s Texture Filters
AMD	Age-related Macular Degeneration
FP	False Positive
IFFT	Inverse Fast Fourier Transform
ILM	Inner Limiting Membrane
RPE	Retinal Pigment Epithelium
GIBS	Global Intensity-Based Features
AIS	Axis Intensity Statistics
EIG	Eigenvalues
LESH	Local Energy-based Shape Histogram
GLCM	Gray-Level Co-Ocurrence Matrix
LBP	Local Binary Patterns
FD	Fractal dimension
DBC	Differential box-counting
SRE	Short Run Emphasis
LRE	Long Run Emphasis
GLN	Gray Level Non-uniformity
RP	Run Percentage
RLN	Run Length Non-uniformity
LGRE	Low Gray Level Run Emphasis
HGRE	High Gray Level Run Emphasis
LDA	Linear Discriminant Analysis
SVM	Support Vector Machine
RBF	Radial Basis Function
kNN	k Nearest-Neighbors
RF	Random Forest
GB	Gradient Boosting

## References

[1] Novo J., Penedo M., Santos J. (2008). Optic disc segmentation by means of GA-Optimized Topological Active Nets. Lect. Notes Comput. Sci. Image Anal. Recognit. ICIAR’08.

[2] De Moura J., Novo J., Ortega M., Charlón P. (2016). 3D retinal vessel tree segmentation and reconstruction with OCT images. Lect. Notes Comput. Sci. Image Anal. Recognit. ICIAR’16.

[3] Wong T., Klein R., Sharrett A., Duncan B., Couper D., Klein B., Hubbard L., Nieto F. (2004). Retinal arteriolar diameters and elevated blood pressure: The Atherosclerosis Risk in Communities Study. Ann. Internal Med..

[4] Ikram M., de Jong F., Bos M., Vingerling J., Hofman A., Koudstaal P., de Jong P., Breteler M. (2006). Retinal vessel diameters and risk of stroke: The Rotterdam Study. Neurology.

[5] Wong T., Klein R., Sharrett A., Schmidt M., Pankow J., Couper D., Klein B., Hubbard L., Duncan B., Investigators A. (2002). Retinal arteriolar narrowing and risk of diabetes mellitus in middle-aged persons. J. Am. Med. Assoc..

[6] Puzyeyeva O., Lam W., Flanagan J., Brent M., Devenyi R., Mandelcorn M., Wong T., Hudson C. (2011). High-resolution optical coherence tomography retinal imaging: A case series illustrating potential and limitations. J. Ophthalmol..

[7] Keane P., Patel P., Liakopoulos S., Heussen F., Sadda S., Tufail A. (2012). Evaluation of age-related macular degeneration with optical coherence tomography. Surv. Ophthalmol..

[8] Baamonde S., Moura J., Novo J., Ortega M. Automatic Detection of Epiretinal Membrane in OCT Images by Means of Local Luminosity Patterns. Proceedings of the International Work-Conference on Artificial Neural Networks—IWANN’17.

[9] Trichonas G., Kaiser P. (2014). Optical Coherence Tomography Imaging of Macular Oedema. Br. J. Ophthalmol..

[10] Bogunovic H., Abramoff M., Zhang L., Sonka M. Prediction of treatment response from retinal OCT in patients with exudative age-related macular degeneration. Proceedings of the Ophthalmic Medical Image Analysis Workshop, MICCAI’14.

[11] Wilkins G., Houghton O., Oldenburg A. (2012). Automated Segmentation of Intraretinal Cystoid Fluid in Optical Coherence Tomography. IEEE Trans. Biomed. Eng..

[12] Roychowdhury S., Koozekanani D., Radwan S., Parhi K. Automated localization of cysts in diabetic macular edema using optical coherence tomography images. Proceedings of the International Conference of the IEEE Engineering in Medicine and Biology Society.

[13] Wieclawek W. Automatic Cysts Detection in Optical Coherence Tomography Images. Proceedings of the International Conference Mixed Design of Integrated Circuits and Systems.

[14] González A., Remeseiro B., Ortega M., Penedo M., Charlón P. Automatic cyst detection in OCT retinal images combining region flooding and texture analysis. Proceedings of the IEEE International Symposium on Computer-Based Medical Systems.

[15] Esmaeili M., Dehnavi A., Rabbani H., Hajizadeh F. (2016). Three-dimensional Segmentation of Retinal Cysts from Spectral-Domain Optical Coherence Tomography Images by the Use of Three-Dimensional Curvelet Based K-SVD. J. Med. Signals Sens..

[16] Wang J., Zhang M., Pechauer A., Liu L., Hwang T., Wilson D.J., Li D., Jia Y. (2016). Automated volumetric segmentation of retinal fluid on optical coherence tomography. Biomed. Opt. Exp..

[17] Xu X., Lee K., Zhang L., Sonka M., Abràmoff M. (2015). Stratified Sampling Voxel Classification for Segmentation of Intraretinal and Subretinal Fluid in Longitudinal Clinical OCT Data. IEEE Trans. Med. Imaging.

[18] Lang A., Carass A., Swingle E., Al-Louzi O., Bhargava P., Saidha S., Ying H., Calabresi P., Prince J. (2014). Automatic segmentation of microcystic macular edema in OCT. Biomed. Opt. Exp..

[19] Lee C.S., Tyring A.J., Deruyter N.P., Wu Y., Rokem A., Lee A.Y. (2017). Deep-learning based, automated segmentation of macular edema in optical coherence tomography. Biomed. Opt. Exp..

[20] Venhuizen F.G., van Ginneken B., Liefers B., van Asten F., Schreur V., Fauser S., Hoyng C., Theelen T., Sánchez C.I. (2018). Deep learning approach for the detection and quantification of intraretinal cystoid fluid in multivendor optical coherence tomography. Biomed. Opt. Exp..

[21] Roy A.G., Conjeti S., Karri S.P.K., Sheet D., Katouzian A., Wachinger C., Navab N. (2017). ReLayNet: Retinal Layer and Fluid Segmentation of Macular Optical Coherence Tomography using Fully Convolutional Network. arXiv.

[22] Lu D., Heisler M., Lee S., Ding G.W., Navajas E., Sarunic M.V., Beg M.F. (2019). Deep-learning based multiclass retinal fluid segmentation and detection in optical coherence tomography images using a fully convolutional neural network. Med. Image Anal..

[23] Chen Z., Li D., Shen H., Mo H., Zeng Z., Wei H. (2020). Automated segmentation of fluid regions in optical coherence tomography B-scan images of age-related macular degeneration. Opt. Laser Technol..

[24] Ronneberger O., Fischer P., Brox T., Navab N., Hornegger J., Wells W.M., Frangi A.F. (2015). U-Net: Convolutional Networks for Biomedical Image Segmentation. Medical Image Computing and Computer-Assisted Intervention—MICCAI 2015.

[25] De Moura J., Vidal P.L., Novo J., Rouco J., Ortega M. (2017). Feature definition, analysis and selection for cystoid region characterization in Optical Coherence Tomography. Procedia Comput. Sci..

[26] Baamonde S., de Moura J., Novo J., Charlón P., Ortega M. (2019). Automatic Identification and Intuitive Map Representation of the Epiretinal Membrane Presence in 3D OCT Volumes. Sensors.

[27] Schuman J.S. (2008). Spectral domain optical coherence tomography for glaucoma (an AOS thesis). Trans. Am. Ophthalmol. Soc..

[28] McKinney W. Data Structures for Statistical Computing in Python. Proceedings of the 9th Python in Science Conference.

[29] Hunter J.D. (2007). Matplotlib: A 2D graphics environment. Comput. Sci. Eng..

[30] Pedregosa F., Varoquaux G., Gramfort A., Michel V., Thirion B., Grisel O., Blondel M., Prettenhofer P., Weiss R., Dubourg V. (2011). Scikit-learn: Machine Learning in Python. J. Mach. Learn. Res..

[31] Oliphant T.E. (2006). A Guide to NumPy.

[32] Chiu S., Li X., Nicholas P., Toth C., Izatt J., Farsiu S. (2010). Automatic segmentation of seven retinal layers in SDOCT images congruent with expert manual segmentation. Opt. Exp..

[33] Dijkstra E. (1959). A note on two problems in connexion with graphs. Numer. Math..

[34] Sarfraz M.S., Hellwich O. (2008). Head Pose Estimation in Face Recognition Across Pose Scenarios. VISAPP.

[35] Kovesi P. (1999). Image features from phase congruency. Videre J. Comput. Vis. Res..

[36] Kovesi P. (2000). Phase congruency: A low-level image invariant. Psychol. Res..

[37] Kovesi P. Phase congruency detects corners and edges. Proceedings of the Australian Pattern Recognition Society Conference: DICTA.

[38] Wajid S.K., Hussain A. (2015). Local energy-based shape histogram feature extraction technique for breast cancer diagnosis. Exp. Syst. Appl..

[39] Wajid S.K., Hussain A., Huang K., Boulila W. Lung cancer detection using Local Energy-based Shape Histogram (LESH) feature extraction and cognitive machine learning techniques. Proceedings of the 2016 IEEE 15th International Conference on Cognitive Informatics & Cognitive Computing (ICCI* CC).

[40] Haralick R., Shanmugam K., Dinstein I. (1973). Textural features for image classification. IEEE Trans. Syst. Man Cybern..

[41] Dalal N., Triggs B. Histograms of oriented gradients for human detection. Proceedings of the Computer Vision and Pattern Recognition, CVPR’05.

[42] Gabor D. (1946). Theory of communication. J. Inst. Electr. Eng..

[43] Haghighata M., Zonouzb S., Abdel-Mottaleba M. (2015). CloudID: Trustworthy cloud-based and cross-enterprise biometric identification. Exp. Syst. Appl..

[44] Al-Kadi O., Watson D. (2008). Texture analysis of aggressive and nonaggressive lung tumor CE CT images. IEEE Trans. Biomed. Eng..

[45] Ojala T., Pietikainen M., Maenpaa T. (2002). Multiresolution gray-scale and rotation invariant texture classification with local binary patterns. IEEE Trans. Pattern Anal. Mach. Intell..

[46] Laws K.I. (1980). Textured Image Segmentation.

[47] Buczkowski S., Kyriacos S., Nekka F., Cartilier L. (1998). The modified box-counting method: Analysis of some characteristic parameters. Pattern Recognit..

[48] Loh H.H., Leu J.G., Luo R.C. (1988). The analysis of natural textures using run length features. IEEE Trans. Ind. Electron..

[49] Nie F., Xiang S., Jia Y., Zhang C., Yan S. Trace Ratio Criterion for Feature Selection. Proceedings of the 23rd National Conference on Artificial Intelligence.

[50] Li J., Cheng K., Wang S., Morstatter F., Robert T., Tang J., Liu H. (2016). Feature Selection: A Data Perspective. arXiv.

[51] Kononenko I. (1994). Estimating Attributes: Analysis and Extensions of RELIEF.

[52] Robnik-Šikonja M., Kononenko I. (2003). Theoretical and Empirical Analysis of ReliefF and RReliefF. Mach. Learn..

[53] Geurts P., Ernst D., Wehenkel L. (2006). Extremely randomized trees. Mach. Learn..

[54] Dasarathy B.V. (1991). Nearest Neighbor (NN) Norms NN pattern Classification Techniques.

[55] Cortes C., Vapnik V. (1995). Support-Vector Networks. Mach. Learn..

[56] Breiman L. (2001). Random Forests. Mach. Learn..

[57] Breiman L. (1998). Arcing classifiers. Ann. Stat..

[58] Friedman J.H. (2001). Greedy function approximation: A gradient boosting machine. Ann. Stat..

